# Heart health for South Asians: improved cardiovascular risk factors with a culturally tailored health education program

**DOI:** 10.1186/s12889-023-15667-y

**Published:** 2023-04-19

**Authors:** Paniz Vafaei, Chi-Mei Liu, Hank Davis, Priyal Patel, Uma Vadlakonda, Seema Pursnani

**Affiliations:** Kaiser Permanente Medical Center Santa Clara, Santa Clara, CA United States

**Keywords:** South asian ethnicity, Cardiovascular risk factors, Cardiovascular outcomes, Culturally tailored health education programs

## Abstract

**Background/Aim:**

The Kaiser Permanente (KP) Northern California Heart Health for South Asians (HHSA) Program is a two-hour educational class that provides culturally relevant lifestyle and dietary recommendations to South Asian (SA) patients, in an effort to reduce their known disproportionate burden of cardiovascular (CV) disease. We evaluated the impact of the HHSA Program on CV risk factors and major adverse CV events (MACE).

**Methods:**

A retrospective cohort study identified 1517 participants of SA descent, ≥ 18 years old from 2006 to 2019. We evaluated the change in risk factors with program attendance (median follow up of 6.9 years) for systolic blood pressure (SBP), diastolic blood pressure (DBP), triglycerides (TG), LDL, HDL, BMI, and HbA1c. We also performed a propensity matched analysis to evaluate differences in MACE including stroke, myocardial infarction (MI), coronary revascularization, and all-cause mortality.

**Results:**

There were significant improvements in DBP, TG, LDL-c, HDL-c, BMI, and HbA1c at one year follow up and sustained improvements in DBP (-1.01mmHg, p = 0.01), TG (-13.74 mg/dL, p = 0.0001), LDL-c (-8.43 mg/dL, p = < 0.0001), and HDL-c (3.16 mg/dL, p = < 0.0001) levels at the end of follow up. In the propensity matched analysis, there was a significant reduction in revascularization (OR 0.33, 95% CI 0.14–0.78, p = 0.011) and mortality (OR 0.41, 95% CI 0.22–0.79, p = 0.008), and a trend towards reduction in stroke.

**Conclusions:**

Our study demonstrates the efficacy of a culturally tailored SA health education program in improving CV risk factors and reducing MACE. The program highlights the importance and value of providing culturally tailored health education in primary CV disease prevention.

## Background

Individuals of South Asian (SA) ancestry from India, Pakistan, Bangladesh, Nepal, Sri Lanka, the Maldives and Bhutan have a disproportionately higher burden of atherosclerotic cardiovascular disease (ASCVD) when compared to other racial and ethnic groups. Nearly 5.4 million South Asians live in the United States and they make up one of the fastest growing minority groups in the country [1]. South Asians have higher hospitalization and mortality rates from ASCVD when compared to other ethnic groups [2–7]. The 2018 and 2019 American College of Cardiology/American Heart Association (ACC/AHA) Cholesterol and Primary Prevention Guidelines now recognize South Asian ancestry as a “risk enhancing factor” [8–9]. The increased risk in South Asians is due, in part, to a disproportionate burden of traditional ASCVD risk factors including diabetes mellitus, dyslipidemia, hypertension, smoking, overweight status and obesity, and the metabolic syndrome. We previously reported that in our Northern California cohort, South Asian ethnicity was associated with a 2 times greater odds of coronary heart disease, as compared to Whites, even after adjustment for traditional risk factors and statin use [10]. Genetic factors, including elevated atherogenic lipid particles (lipoprotein (a) and apolipoprotein B) in South Asians, and unmeasured lifestyle factors, may also contribute to this heightened risk [11–13].

In communities densely populated by South Asians, health education and primary prevention efforts exist, but cardiovascular outcomes from these efforts are seldom reported. Kaiser Permanente (KP) Northern California established the Heart Health for South Asians (HHSA) Program in 2006 in a primary prevention effort to reduce the known health disparity in ASCVD risk in South Asians. The HHSA program is an ongoing two-hour educational class that is offered at Kaiser Permanente Santa Clara, California that provides detailed education regarding risk factors for ASCVD and culturally relevant lifestyle recommendations regarding diet, exercise, and stress reduction to patients who self-identify as being of South Asian descent. The first hour is run by an internal medicine and/or cardiovascular medicine trained physician, focused on education of standard ASCVD risk factors and ASCVD diagnosis and treatment. Education was specifically provided on risk factors unique to South Asians, including attention to the greater prevalence of metabolic syndrome and its components: lower HDL-cholesterol, greater waist to hip ratio, and pre-diabetes. The second hour is led by a dietitian and health educator, focused on healthy South Asian cooking and meal choices for the family, with attention to reading food labels in South Asian grocery stores and guidance regarding types of oil use in cooking, Attention was also given to the myth of all vegetarianism being “healthy”, with education regarding healthy and unhealthy vegetarian food choices and a shift in focus to a whole food, plant based diet. The pillars of lifestyle medicine (sleep hygiene, substance abuse cessation, healthy eating, exercise, and stress reduction) were also incorporated int o the teaching program Participants are requested to have routine blood test screening for dyslipidemia and diabetes prior to class attendance and follow up is typically ensured with their primary care physician thereafter, with the option for continued health education/dietitian involvement. Members have the option to fill out an anonymous survey regarding health attitudes and lifestyle practices.

Our current study aims to evaluate the health of members who attended the class by compilation of self-reported data acquired from the health survey and evaluation of individual cardiovascular risk factors prior to and following class attendance through retrospective electronic health record review. In a propensity matched analysis, we also aim to evaluate the association of participation in HHSA on the development of later major adverse cardiovascular events (MACE).

## Methods

We performed a retrospective cohort study using participants who attended the HHSA program from 2006 to 2019, with age 18 or older and self identifying as South Asian ethnicity. Participants were excluded if electronic health record follow up was not available in 2019. Participants with existing ASCVD were excluded based on review of International Classification of Diseases (ICD)-10 and 11 codes. The following data were collected at three time points, within 12 months prior to program participation, the earliest of 6–24 months following program participation, and the last available follow up, up to December 2019: systolic blood pressure (SBP), diastolic blood pressure (DBP), triglycerides (TG), low density lipoprotein (LDL-c), high density lipoprotein (HDL-c), body mass index (BMI), and glycated hemoglobin (HbA1c). Descriptive statistics (frequency, mean, median, proportions) for the demographic and clinical characteristics were calculated using the chi-squared test for categorical variables and the t-test for continuous variables. A paired t-test was used to evaluate differences in these risk factor values across the early and late follow up time periods.

A propensity matched analysis was conducted using the MatchIt R program, with HHSA participants matched 1:1 to a group of KP members who did not enroll in the HHSA program, matched for ethnicity, gender, age, and presence of hypertension, diabetes, and/or hyperlipidemia. Odds ratios for MACE, defined as stroke, myocardial infarction (MI), coronary revascularization, and all-cause mortality, were calculated for each group and Fisher’s exact test used for statistical comparison.

An optional risk profile survey was administered at initial enrollment and included the following data: self-reported family history of early coronary artery disease (CAD), secondhand smoke exposure, physical activity level, consumption of whole grains, vegetables, fat, meat, sodium, caffeine, alcohol, weight change since age 21, and self-reported emotional wellbeing. Descriptive statistics of survey data were calculated using chi-squared test for categorical variables and t-test for continuous variables.

Stata 15.0 and R were used for statistical analysis.

## Results

We identified a total of 1,517 participants who attended HHSA between 2006 and 2019 (between 1 and 13 years follow up, with a median follow up 6.9 years), with a mean age of 42.5 years and 66% male. Of the cohort, 16% had type 2 diabetes mellitus, 22% had hypertension and the mean body mass index was 26 kg/m2. Baseline demographic and cardiovascular risk factor data for the cohort is shown in Table 1.

We evaluated the difference in individual cardiovascular risk factors including SBP, DBP, TG, LDL-c, HDL-c, BMI, and HbA1c prior to and post program attendance, at one year follow up and at the end of the follow-up period. At one year follow-up, there were improvements in DBP (-0.63mmHg, p = 0.006), TG (-25.92 mg/dL, p = < 0.0001), LDL-c (-12.66 mg/dL, p = < 0.0001), HDL-c (0.36 mg/dL, p = 0.02), BMI (-0.38 kg/m2, p = < 0.0001), and HbA1c (-0.22%, p = < 0.0001). At the end of available follow-up, there were sustained reductions in DBP (-1.01mmHg, p = 0.01), TG (-13.74 mg/dL, p = 0.0001), LDL-c (-8.43 mg/dL, p = < 0.0001), and HDL-c (3.16 mg/dL, p = < 0.0001) levels. There were also increases in recorded SBP (2.72mmHg, p = < 0.0001) and HbA1c (0.12%, p = 0.005) [Table 2].

**Fig. 1:**
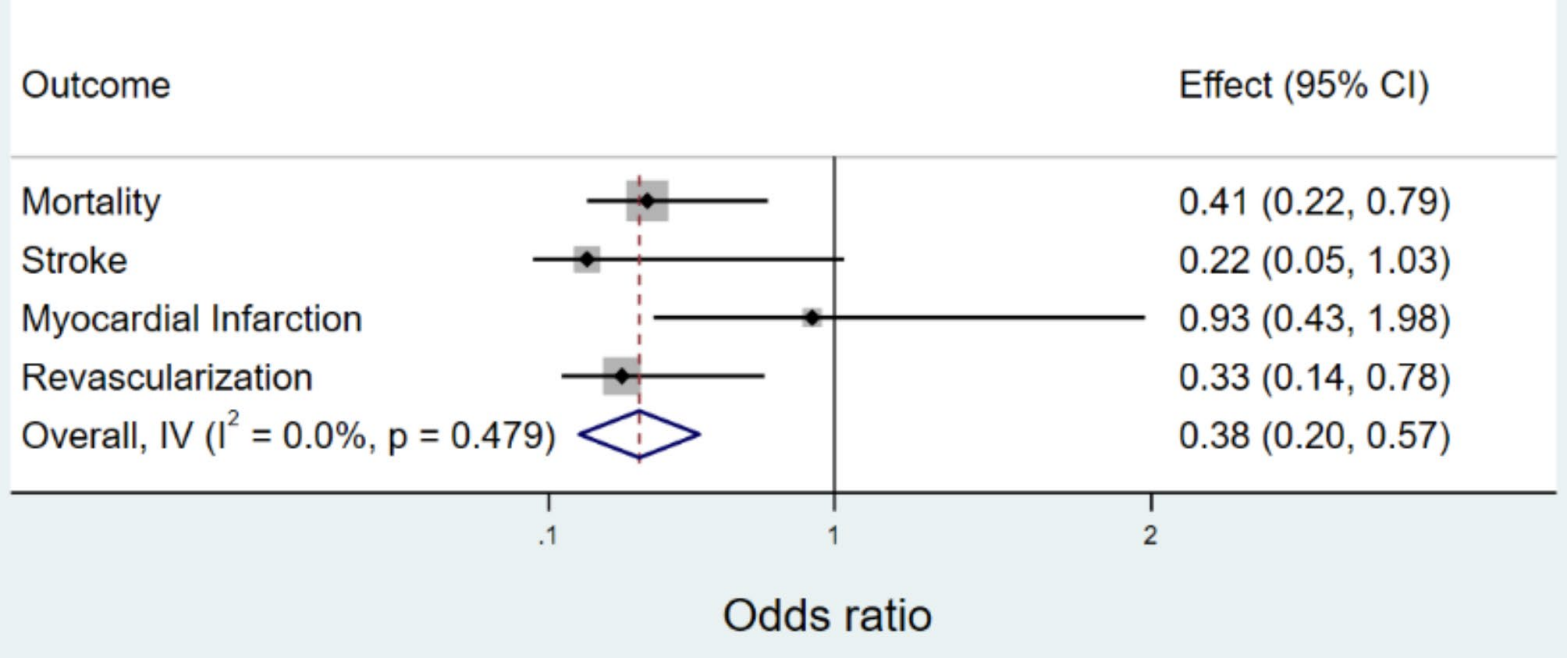
Odds of MACE in participants of HHSA versus a propensity matched group that did not participate in HHSA Propensity matched analysis showing the odds of MACE in patients who attended versus those who did not attend the HHSA program

In the propensity matched analysis, HHSA participation was associated with a reduction in odds of revascularization (OR 0.33, 95% CI 0.14–0.78, p = 0.011) and all-cause mortality (OR 0.41, 95% CI 0.22–0.79, p = 0.008). There was also a trend toward reduction in stroke (OR 0.22, 95% CI 0.05–1.03, p = 0.054). There was no statistically significant difference in the odds of incident MI (OR 0.93, 95% CI 0.43–1.98, p = 0.85) [Fig. 1].

A total of 383 surveys were compiled, with mean age of 45.3 years (range 22–80) and 66.8% male gender. The results of the survey are outlined in Table 3. A total of 44.1% respondents reported a family history of early CAD (p = 0.037 across age decade); 6.6% reported secondhand smoke exposure. While only 24.4% reported at least 5 days per week of aerobic exercise, 24.7% reported less than 30 min of aerobic exercise per week (p = 0.027 across age decade). Self-reported dietary habits were notable for 48.7% of participants reporting eating two or less daily servings of fruits or vegetables (p = 0.001 across age decade), 54.7% being predominantly vegetarian, and 98.9% drinking one or less alcoholic beverage per day. Weight gain greater than 40 pounds since age 21 was reported in 21.1% of participants (25.6% of women and 18.8% of men). Emotional well-being was assessed by burden of negative emotions, with 81.7% of participants reporting no negative emotions each day.

## Conclusion

In our retrospective cohort study, we evaluated participants who attended the HHSA program between 2006 and 2019 in a geographical location densely populated by South Asians (Santa Clara, California). Our findings demonstrate that participation in a culturally tailored South Asian primary prevention health education program is associated with long-term improvements in LDL-c, HDL-c, and TG levels and short-term reductions in DBP, BMI, and HbA1c. Participation in HHSA was also associated with lower odds of MACE, as compared to a propensity matched South Asian group that did not attend the HHSA program.

Culturally tailored lifestyle and behavioral intervention programs have shown mixed promise in improving cardiovascular risk profiles in this high-risk ethnic group. The South Asian Heart Lifestyle Intervention (SAHELI) study was a single center, randomized controlled trial that evaluated the efficacy of a culturally tailored, community-based, lifestyle intervention program for 63 South Asians at-risk for ASCVD in Chicago. The intervention included 6 interactive group classes focusing on physical activity, diet, weight and stress management while the control group received printed education material. There were no significant differences in the primary outcomes including change in physical activity and dietary saturated fat intake. The intervention group had more significant weight loss and a greater decrease in HbA1c, while no significant changes were seen in blood pressure or total cholesterol [14].

The PROgramme of Lifestyle Intervention in Families for Cardiovascular risk reduction (PROLIFIC) study was a randomized controlled trial evaluating the efficacy of a family-based intervention for cardiovascular risk reduction in individuals with a family history of premature CAD in Kerala, India. The intervention group received screening for CV risk factors, structured family-based lifestyle interventions, linkage to a primary care facility and active follow up for adherence. The study showed that the odds of achieving the primary outcome, which was defined as an improvement in blood pressure, fasting glucose, LDL-c reduction, or tobacco cessation was 2.2 times higher in the intervention group than in the usual care group [15].

The South Asian HeArt Risk Assessment (SAHARA) trial was a randomized controlled trial that assessed the efficacy of a culturally tailored digital health intervention on MI risk in a primary prevention South Asian population in Canada. Regular emails or text messages focused on improving diet and exercise were applied to the intervention group and an MI risk score, which included the objective components of blood pressure, waist to hip ratio, hemoglobin A1C level, and ratio of apolipoprotein B to apolipoprotein A, was evaluated at baseline and at one year follow up. While the MI scores were desired by participants and motivated behavioral changes, no significant change was found in the MI score at follow up [16].

These randomized studies suggest a role for lifestyle-based interventions in aiming to reduce ASCVD risk in a high-risk ethnic group. In our study, we hypothesize that changes in risk factors and the odds of MACE seen with HHSA participation is likely due to unmeasured intrinsic motivational factors, health attitudes, and lifestyle factors. Our study is limited by its design as a retrospective study and without a randomized intervention. Strengths of our study include the large cohort and use of an integrated health delivery system for long-term follow up. Future efforts aim to focus on understanding health motivation and delivery of lifestyle medicine to South Asians.

**Table 1:** Baseline characteristics of participants in the HHSA Program between 2006–2019

Baseline Characteristics	Mean (SD) or Frequency
Age	42.5 (11.0)
Males	66.3%
Smoking history: active / former / never	2.2% / 10.1% / 81.3%
Diabetes Mellitus	15.9%
Hypertension	22.0%
Chronic kidney disease	0.9%
Body mass index (kg/m2)	26.3 (3.8)
Systolic blood pressure (mmHg)	119.9 (14.0)
Diastolic blood pressure (mmHg)	72.6 (10.0)
LDL-cholesterol (mg/dL)	116.5 (36.2)
HDL-cholesterol (mg/dL)	43.0 (10.0)
Triglycerides (mg/dL)	173.8 (170.7)

**Table 2:** Cardiovascular risk factor changes at early and late follow up time points for participants in HHSA

Risk Factor	One Year Follow Up		End of Follow Up (median follow up 6.9 years)	
	Change in Mean	p-value	Change in Mean	p-value
**Systolic blood pressure (mmHg)**	0.09	0.78	2.72	< 0.0001
**Diastolic blood pressure (mmHg)**	-0.63	0.006	-1.01	0.01
**Triglycerides (mg/dL)**	-25.92	< 0.0001	-13.74	0.0001
**LDL-c (mg/dL)**	-12.66	< 0.0001	-8.43	< 0.0001
**HDL-c (mg/dL)**	0.36	0.02	3.16	< 0.0001
**BMI (kg/m2)**	-0.38	< 0.0001	0.10	0.22
**HbA1c (%)**	-0.22	< 0.0001	0.12	0.005

**Table 3:** Select survey data regarding lifestyle risk factors from participants in HHSA

Survey Question	Percentage
Secondhand smoke exposure	6.6%
Greater than 40 pound weight gain since age 21	21.1%
Meeting exercise recommendations ( > = 5 days/week)	24.4%
Participating in less than 30 min of exercise per day	24.7%
Vegetarian	54.7%
Intake of at least 2 servings of fruits or vegetables per day	48.7%
Consumption of less than one alcoholic beverage per day	98.9%

## Data Availability

The data that support the findings of this study are available from Kaiser Permanente but restrictions apply to the availability of these data, which were used under license for the current study, and so are not publicly available. Data are however available from the corresponding author (Dr. Pursnani) upon reasonable request and with permission of Kaiser Permanente.
